# Artificial Neuron Based on Integrated Semiconductor Quantum Dot Mode-Locked Lasers

**DOI:** 10.1038/srep39317

**Published:** 2016-12-19

**Authors:** Charis Mesaritakis, Alexandros Kapsalis, Adonis Bogris, Dimitris Syvridis

**Affiliations:** 1 Department of Informatics & Telecommunications, National and Kapodistrian University of Athens, Panepistimiopolis Ilisia 15784, Athens, Greece; 2 Department of Informatics, Technological Educational Institution of Athens, Aghiou Spiridonos, 12210 Egaleo, Athens, Greece

## Abstract

Neuro-inspired implementations have attracted strong interest as a power efficient and robust alternative to the digital model of computation with a broad range of applications. Especially, neuro-mimetic systems able to produce and process spike-encoding schemes can offer merits like high noise-resiliency and increased computational efficiency. Towards this direction, integrated photonics can be an auspicious platform due to its multi-GHz bandwidth, its high wall-plug efficiency and the strong similarity of its dynamics under excitation with biological spiking neurons. Here, we propose an integrated all-optical neuron based on an InAs/InGaAs semiconductor quantum-dot passively mode-locked laser. The multi-band emission capabilities of these lasers allows, through waveband switching, the emulation of the excitation and inhibition modes of operation. Frequency-response effects, similar to biological neural circuits, are observed just as in a typical two-section excitable laser. The demonstrated optical building block can pave the way for high-speed photonic integrated systems able to address tasks ranging from pattern recognition to cognitive spectrum management and multi-sensory data processing.

The enormous technological leaps in terms of hardware and software development of the last decades have provided the means to address a vast class of problems that require extensive computational power. These approaches mainly involve optimized von-Neumann architectures that despite their extensive deployment and efficiency still offer limited performance in addressing tasks like pattern recognition, machine vision, natural language processing, motion control for autonomous robotics, and decision making^1^.

Neuro-inspired computational paradigms have risen as an alternative means to provide an energy efficient and robust solution to this age-old problem, by emulating basic biological neural functions and by taking advantage of characteristics like the analog-digital signal representation and colocation of memory and processing^2^,^3^. An interesting subset of neuro-mimetic architectures are the ones that exploit the spiking encoding scheme, which is a digital-analog hybrid that allows information to be represented both in time and space^4^. This encoding strategy offers increased noise resilience^5^ and potential improvements in terms of computational efficiency^6^. Towards this direction, newly emerging electronic circuits like CMOS-neurons^7^ and interconnections based on memristors^7^,^8^ have garnered success in various applications by providing enhanced performance, low power consumption and exceptional miniaturization capabilities. However, the upscaling of electronic neuro-mimetic systems requires an extensive number of nodes and inter-neuron connections. A straightforward increase is not easily achievable due to inherent impediments like the detrimental effects of electromagnetic interference and the fundamental bandwidth fan-in tradeoff 9,10,11,12; thus forcing the adoption of address-event representation schemes that allow higher virtual connection densities, however at the expense of bandwidth^12^.

Alternatively, photonic technologies can provide an auspicious platform for the development of high-speed spike-processing schemes due to inherent advantages like ultra-high bandwidth, low propagation losses, high wall-plug efficiency^12^,^13^,^14^,^15^,^16^, and inherent parallelism^17^,^18^. The behavior of the dynamics observed in biological spiking neurons shares many similarities with various photonic components, with the additional advantage that the response time of photonic systems is orders of magnitude lower than their biological counterparts^19^. These advantages can benefit an ever-growing number of applications that require high-speed brain-inspired processing like motion control, multi-sensory processing, cognitive processing of the RF spectrum and fast adaptive control^12^.

The above advantages have been identified decades ago and several optical approaches have been already proposed that incorporate bulk setups and non-linear crystals^1^,^20^,^21^,^22^. A significant effort has been put into the realization of photonic integrated neurons, based on various semiconductor devices, in order to minimize the neuron’s footprint and energy consumption. The use of semiconductor devices is also favorable due to their rich dynamics that can be triggered via optical or electrical injection. Depending on the underlying physical mechanism present in each case, these systems can emulate effects similar to neural excitability or to periodic spiking^12^,^23^. Photonic integrated neurons include two-section (gain and saturable absorber) lasers^19^,^24^,^25^,^26^,^27^,^28^,^29^,^30^, semiconductor micro-rings and disks^31^,^32^,^33^, single section quantum-dot lasers^34^, nano-cavities based on 2D photonic crystals^35^,^36^, lasers subject to optical injection^37^,^38^,^39^ or feedback^40^,^41^,^42^ and polarization switching VCSELs^43^,^44^. Despite the merits of each implementation and the various levels of isomorphism achieved, the up-to-date approaches fail to all-optically emulate both types of biological neurons (excitatory and inhibitory). In order to highlight the importance of both excitatory and inhibitory functions, the fundamental operational properties of biological neurons are briefly discussed in the subsequent paragraphs.

A single biological neural cell can be described, from an operational point of view, as a threshold driven leaky integrator that can be impelled to activation, or suppression, depending on the inputs that the neuron receives, following an all-or-nothing principle. Although these inputs are a prerequisite condition for neural excitation, the temporal characteristics of the neuron’s response are solely governed by its internal dynamics^45^. The biological neurons can be classified into two basic categories. The first, defined as excitatory neurons (Fig. 1a), includes nodes that upon activation tend to force all the connected neurons into a firing activity. The second category (Fig. 1b), defined as inhibitory neurons, includes nodes that although activated through the same stimulus as excitatory neurons, when triggered, they emit pulses that tend to suppress activity in all connected nodes^46^.

The existence of these two categories of neurons, as well as their interconnection in well-defined motifs, forms neural patterns that are crucial for the realization of basic motor operations and for limiting the total neural firing activity^47^. Furthermore, these motifs are interconnected in order to form more complex structures that are linked to advanced cognitive processes^47^,^48^. A characteristic example of such a motif is the feedback inhibition structure presented in Fig. 1c 49. Previous all-optical photonic implementations targeted at the reproduction of only one of the abovementioned neuron types (Fig. 1a). In those cases, all activated neurons emitted only excitation signals, thus failing to emulate the inhibitory regime of operation described in Fig. 1b. Recently, a numerical investigation of an inhibitory-excitatory neuron based on a two-section laser was presented^50^. In that work, the output of each laser neuron is fed to a photodiode that in turn pushes the subsequent neuron towards to, or away from, activation. Although this approach is interesting, it is based on electro-optical conversion, thus is subject to bandwidth limitations and leads to increased system complexity.

Here we provide experimental results showing that an integrated InAs/InGaAs Fabry-Perot quantum-dot mode-locked laser can realize both types of neurons simply by choosing the appropriate biasing conditions. In our case, inhibition and excitation are associated with waveband switching effects triggered solely through optical injection from another optical neuron. The waveband related excitation spikes are of nanosecond duration and manifest as an envelope signal enclosing the short mode-locked pulses. In addition, the replication of frequency-response effects, similar to biological neural circuits, are observed just as in a typical two-section excitable laser.

## Results

The devices used for the realization of the artificial neuron were 2 mm long two-section InAs/InGaAs quantum-dot passively mode-locked lasers (see methods). The unique “atom in a box” behavior of the quantum-dots enables single or dual emission from two discrete wavebands, using radically lower injection current compared to quantum-well based devices^51^. We associate pulses from the lower energy band (ground state) as the neural excitation signals, whereas pulses from the first excited state correspond to inhibitory signals. At this point, we would like to clarify that the excited state emission of a typical quantum dot laser should not be confused with the excited state in the neural sense. The excitatory or inhibitory signals were provided by another identical quantum-dot laser, which was incorporated with the neuron-acting laser in a typical master-slave injection-locking scheme (Fig. 2a). Prior to the experimental realization of the system, each device was electro-optically characterized in order to identify the optimum biasing conditions and investigate performance in terms of optical power, pulse width and waveband of emission (see supplementary information).

The excitatory neuron is realized when the laser is biased to exhibit single excited state emission (I ≈ 1.2I_th_ and V_abs_ = −5 V). An optical isolator prevents mutual coupling and self-feeding and a waveband filter eliminates neuron emission in the absence of an input signal (Fig. 2b). The inhibitory neuron is biased at the same current as the excitatory one, whereas a reduction in the reverse voltage (V_abs_ = −4 V) allows single ground state emission. An isolator and a waveband filter that blocks ground state emission are used similarly to the excitatory neuron (Fig. 2c).

### Excitatory Neuron

The basic operation of the excitatory neuron is visualized with the help of the optical transitions presented in Fig. 3a. When ground state pulses are injected (excitation signal), the device is forced to partially suppress excited state emission and a significant increase in the order of 25 dB in the ground state band is achieved (excitatory signal) (Fig. 3a). Complementary, if the neuron is fed with excited state pulses (inhibition) only a minor enhancement in the excited state emission is observed, while spontaneous emission from the ground state is further suppressed, pushing the device away from ground state threshold (neural inhibition). Consequently, the neuron, similarly to previous implementations, is driven towards, or away from, activation (ground state lasing) depending on the wavelength of the external stimulus.

Taking also into consideration the mode locking mechanisms present in our case, the RF spectrum is also investigated as a function of the injection strength for the aforementioned regime. More specifically, in the absence of input, the neuron produces pulses with a repetition frequency of 20.01 GHz (excited state) and an extinction ratio of approximately 26 dB (Fig. 3b). In case that an excitation signal (ground state pulses) is injected, the device is driven to a dual mode-locking regime. The repetition frequency of the laser at the ground state is identical to the input, implying injection locking. If injection strength is maximized, the excited state emission is completely suppressed and the significant broadening of the RF peak provides indications of a possible shift to a coherence collapse regime^52^. On the other hand, when feeding the neuron with an inhibitory signal at the maximum injection level, no significant spurious tones in the RF spectrum can be monitored.

The waveband transitions observed in the excitatory regime can be related to two phenomena. The direct variation of the free-carrier population at each state (ground-excited state) due to the optical injection and indirectly, through the quantum-dot related intra-band dynamics. More specifically, the injection of optical power in the excited state reduces the free carrier density also reducing the occupation probability at this band. This, in turn, inhibits the relaxation of carriers in the ground state^53^, thus damping the gain, equivalently increasing the ground state’s lasing threshold. On the other hand, the injection of ground state pulses increases the photon density in this band and consequently allows lasing in a similar way as an increase in the injection current. In the experiments, we observed that the excited state’s RF peak remains mainly unaffected even at elevated injection strength compared to the ground state injection case, where coherence collapse can be recorded. This fact can be attributed to the lower linewidth enhancement factor associated with the excited state band^54^,^55^.

### Inhibitory neuron

**Figure 1:**
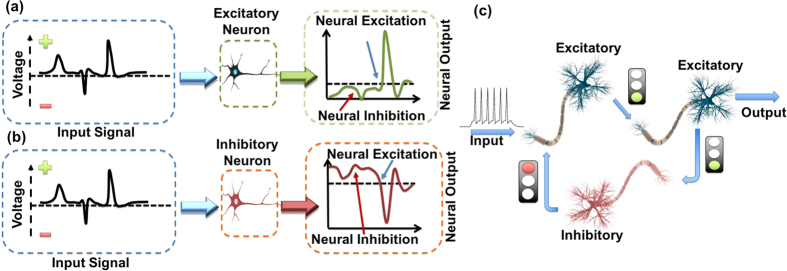
(**a**) Schematic representation of the operation of a single excitatory neuron. (**b**) Schematic representation of a single inhibitory neuron that when activated emits inhibitory signals to subsequent nodes. (**c**) A schematic of a fundamental neural motif (feedback inhibition) that consists of two excitatory and one inhibitory neurons.

**Figure 2:**
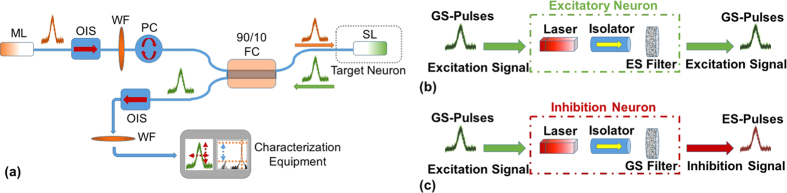
(**a**) The experimental setup: ML/SL corresponds to master/slave lasers, OIS to optical isolator, PC to polarization controller, WF to waveband filters while FC a fiber coupler. (**b**) Schematic representation of the basic operation of an excitatory neuron that includes a filter blocking emission from the excited state (ES) in the absence of an input signal. (**c**) Schematic representation of the basic operation of an inhibition neuron that includes a filter blocking emission from the ground state (GS) in the absence of an input signal.

**Figure 3:**
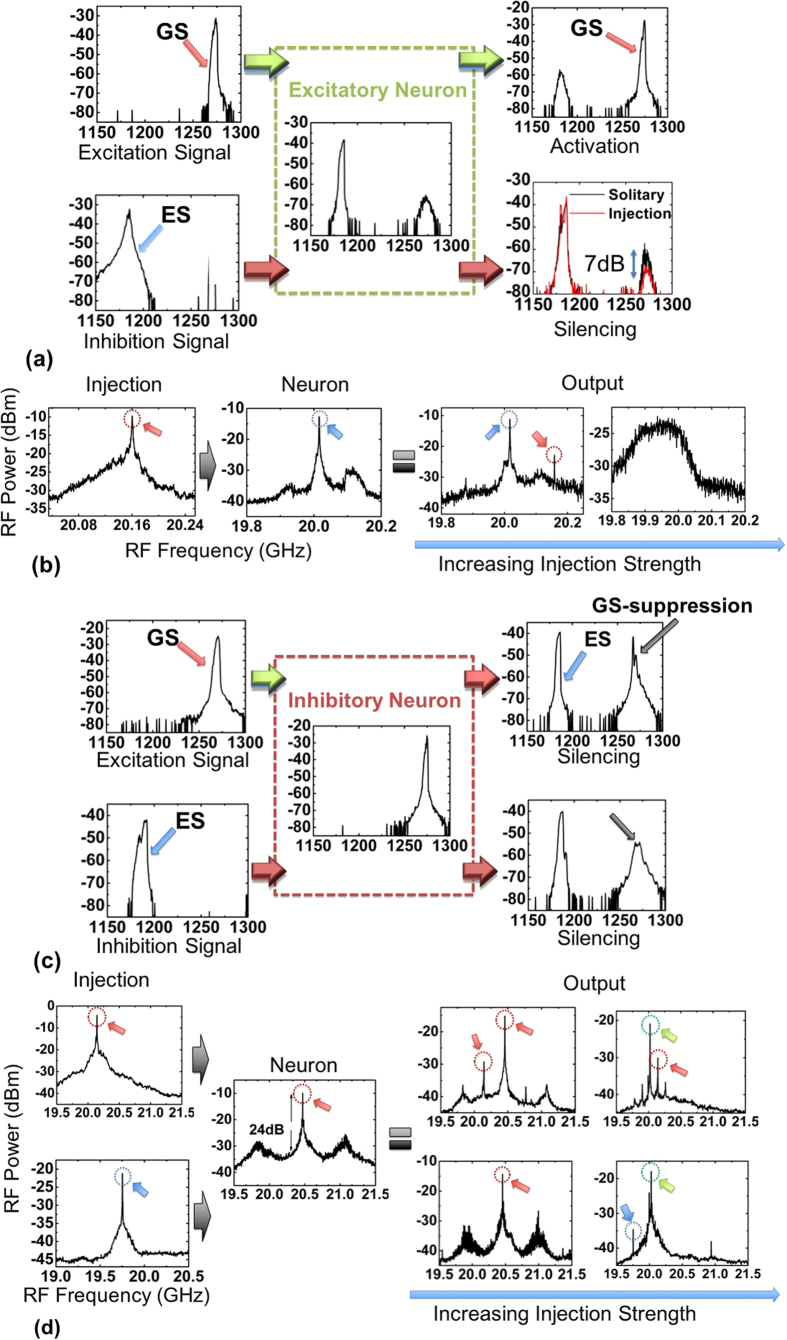
(**a**) Optical spectra that demonstrate the switching behavior under external optical injection of an excitatory neuron. (**b**) Evolution of RF spectrum for weak and strong injection for an excitatory neuron under ground state injection. (Red circles highlight RF peaks that correspond to ground state mode locking, blue to excited state. (**c**) Optical spectra that demonstrate the switching behavior under external optical injection of an inhibitory neuron. (**d**) Evolution of the RF spectrum for an inhibitory neuron under both stimuli (excitation-inhibition). Green circles are associated to excited state mode-locking but with independent of the neuron-injection repetition frequency.

The basic operation of the inhibitory neuron can be identified in the optical transitions presented in Fig. 3c. When ground state pulses are injected in the neuron (excitation), an unexpected suppression of ground state emission is observed (ΔP_opticalGS_ ≈ −15 dB), accompanied by the initialization of strong excited state emission (inhibitory signals) (Fig. 3c). In contrast, injection of pulses originating from the excited state (inhibition), evokes a strong suppression of ground state emission exceeding −22 dB. This operational regime does not have an equivalent in previous all-optical implementations. Specifically, upon activation, the neuron emits inhibitory signals, forcing neural silencing similar to Fig. 1b.

Regarding the RF spectrum against injection strength, in the absence of an external input, the neuron exhibits a clear peak (20.47 GHz) (Fig. 3d) that corresponds to strong mode-locking from the ground state. This is accompanied by weak sidebands, with a frequency spacing in the order of 600 MHz, which are associated to Q-switching due to the bias proximity to excited state lasing threshold. When the neuron is subject to an excitation stimulus (20.14 GHz), which corresponds to optical pulses from the ground state, the following behavior is recorded. Under weak injection, two main peaks can be identified corresponding to ground state pulses of the two lasers. If the injection strength is further increased, two peaks can still be observed, however the first peak (20.02 GHz repetition frequency) corresponds to clear mode locking from the excited state (green circle of Fig. 3d), whereas the second matches to the repetition frequency of the input signal (red-circle). Going to the excited state input case, the situation is as follows. Under weak injection at the excited state, the Q-switching instabilities are enhanced, whereas for stronger injection the RF peak that corresponded to ground state mode locking is utterly suppressed.

The physical mechanism of this abrupt ground state suppression, observed in this case, does not stem from typical free-carrier variation as in the excitatory case. In particular, injection from the ground state causes a significant increase in the homogeneous broadening, due to the increase in the intracavity energy and temperature that leads to a ground state quenching effect^53^,^56^. The increase of the homogeneous broadening allows the redistribution of free carriers to non-lasing dots and the excited state. Conversely, this effect is not observed under excited state injection, where only variations in the free-carrier density govern waveband switching. This asymmetry in waveband behavior has been recently predicted numerically in quantum dots, and has been attributed to the different levels of hole depletion at each energy-band^57^. It must be emphasized that the unavoidable repetition frequency mismatch (up to 300 MHz – see Fig. 3d) between the two lasers, when they are lasing in different emission states, did not hinder the stability of waveband transitions, implying that the excitability of the system is governed by the injected power as it will be shown in the following paragraphs.

Summarizing, in the absence of input, the output of both types of neurons is blocked due to the existence of the waveband filters at the system’s output. For the excitatory case, if an excitation signal (ground state) is injected, then the neuron is activated resulting to the onset of excitation signal emission (ground state). In the symmetric case, if an inhibitory signal (excited state) is injected then the neuron is driven away from ground state emission (inhibition). For the inhibitory neuron, if an excitation signal (ground state) is injected, the neuron is again activated, but contrary to previous implementations, emits inhibitory signals (excited state).

**Figure 4:**
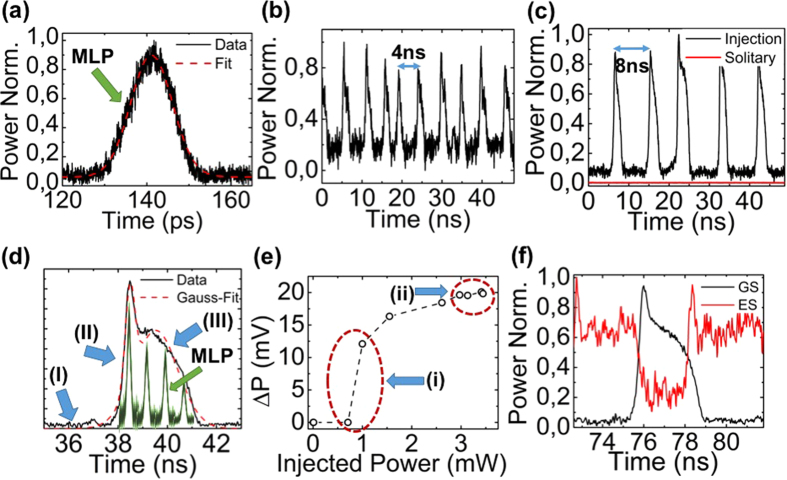
(**a**) Second harmonic autocorrelation trace alongside Gaussian fit for the injected mode-locked optical pulse exhibiting a duration of 10.6 ps. MLP stands for mode-locked pulse. (**b**) Time trace of an excitatory neuron under pulsed ground state injection of a master laser with 20 GHz repetition frequency and (**c**) with 10 GHz repetition frequency. (**d**) Magnification of a single excitation spike (black-solid) highlighting the different regimes (arrows) the artistic inclusion of MLPs (green), demonstrates the simultaneous existence of waveband related spikes and mode-locked pulses. (**e**) Power difference between the maximum and minimum of the neuron’s output (excitation amplitude) versus the injected average power from the master laser. (**f**) Time traces for the ground (black) and excited (red) state under ground state injection.

In the above paragraphs, the waveband switching behavior of the devices for different inputs was confirmed through the evaluation of the optical spectra, whilst the investigation of the RF spectra guaranteed the preservation of mode locking throughout these transitions. Nonetheless, in order to confirm neural excitability and investigate the temporal characteristics of each waveband related excitation (spike), a time transient analysis is required. In this respect, optical power time-traces were recorded using a photodiode with a bandwidth of 10 GHz and a real-time oscilloscope with 40 Gsa/s data acquisition capabilities. It is worth mentioning that through this experimental setup (real-time oscilloscope) the generated mode-locked pulses could not be recorded due to their short duration. Without loss of generality, we have biased the neuron in an excitatory regime (excited state emission) and picosecond pulses (Δt = 10.6 ps–Fig. 4a) from the ground state were injected in the system with a repetition frequency of 20 GHz (neural excitation). Using optical filters, time traces from each waveband were recorded at the neuron’s output. In Fig. 4b a time trace corresponding to the ground state band is presented. Despite the fact that the neuron receives constantly pulses from the master laser and is injection locked (see Fig. 3a,b), the ground state emission exhibits low-frequency pulsations (spikes) with a period of T ≈ 4 ns. This implies that switching between wavebands is not stable but the neuron periodically shifts back to single excited state emission.

Aiming to cross-validate this observation, a different laser source has been used as the neuron’s input, exhibiting half the repetition frequency (10 GHz) and emitting pulses with different temporal characteristics but the same peak power. Under this excitation, the neuron was injection locked to the second harmonic of the input and identical periodic pulsations (spikes) were also recorded, but with half the frequency (T ≈ 8 ns) (Fig. 4c). In the absence of input pulses (Fig. 4c-red trace), the power at the ground state is minimized and no evidence of spikes could be monitored. In Fig. 4d, we zoomed-in in a single excitation of Fig. 4c, and performed Gaussian fitting, assuming two peaks. Three discrete regimes are identified. In (Fig. 4d I), incoming mode-locked pulses from the ground-state are accumulated in the target neuron^30^. After a specific threshold, a rapid increase of the ground state emission is observed with a rise time of 420 ps (Fig. 4d II). The spike reaches a peak and power decreases following a significantly slower process, t = 1.4 ns, without being affected by the incoming mode-locked pulses (Fig. 4d III). Furthermore, in Fig. 4d we have included an artistic representation of the mode-locked pulses so as to highlight that the neural-spike is an envelope of the underlying mode-locked pulses.

Aiming to further confirm the excitability of the target neuron, the approach of M. A. Larotonda *et al*.^26^ has been followed. Employing the same experimental conditions as in Fig. 4c, we varied the average optical power of the injected signal and recorded the amplitude of the excitable spikes (Fig. 4e). It can be seen that when increasing the injected power from 0.8 mW to 1.1 mW an abrupt transition occurs from a no pulsation (ΔP = 0) to a spiking state (Fig. 4e i). The amplitude of the output spikes increases with the incoming power until 2.7 mW, whereas from this point and up to the maximum injected power (P_in_ = 3.5 mW), no change in the spike amplitude was measured (Fig. 4e ii). Furthermore, the complementary transitions between the two wavebands during excitation is demonstrated in Fig. 4f using an optical coupler and two waveband filters.

The observations of Fig. 4b–f support the claim that our approach fulfills the three fundamental criteria of excitability^58^. Upon activation, a large excursion from equilibrium is recorded. In our case, this effect can be traced by the existence of a steep activation threshold presented in Fig. 4e–i. Secondly, the system returns to equilibrium (initial waveband of emission) after activation, followed by a refractory period (Fig. 4b,c), where no activation is achieved. In our approach, the Fig. 4b,c highlight this fact. In particular, even though mode-locked pulses are constantly injected in the neuron, after each nanosecond spike the system returns to the initial waveband of emission and it is not affected by the additional mode-locked pulses (refractory period of 4 ns–8 ns). In the absence of input, the system rests at a stable equilibrium (Fig. 4b,c – red trace). An additional evidence that substantiates neural-like excitability is the fact that similar to previous works^58^ we have observed that the amplitude of the spikes is relatively independent of the incoming power (Fig. 4e–ii).

The underlying physical mechanism in our case is similar to two-section lasers biased below threshold, where a transition to a spiking regime is achieved through a homoclinic saddle node bifurcation^12^,^58^. The gain section is a temporal integrator, while the saturable absorber acts as a threshold detector^12^,^19^,^24^. In our case, although the device is lasing (ground/excited state emission), both regimes of operation are characterized by the fact that one of the two states is in a sub-threshold condition. In the excitatory case, single excited emission is observed but the ground state is below/near threshold, opposite to the inhibitory case.

The aforementioned results could be interpreted by adapting the gain-absorber dynamics analysis to the waveband transitions of quantum-dot lasers mentioned in the previous paragraphs. For example, in the case of exciting an excitatory neuron (ground state input in a predominantly excited state emitting laser), the energy of the incoming pulses induces a gradual carrier decrease alongside an increase in the photon density in the ground state band. The intracavity energy surpasses a certain threshold and the absorber is bleached resulting in a picosecond intracavity loss decrease, whose timescale is solely dictated by the reverse applied voltage. Despite the continuous accumulation of incoming pulses, a refractory period follows, during which the neuron’s gain recovers, however at a significantly slower time scale^24^. As a two-section laser system in its core, the proposed scheme renders the replication of the frequency response effects observed in biological neurons plausible. This isomorphism is implied when comparing Fig. 4b,c. In these two cases, the external stimuli differ in terms of repetition frequency (from 20 GHz to 10 GHz), but share the same peak power (P_peak_ ≈ 60 mW). This variation in the received power results in doubling the inter-spike period (from 4 ns to 8 ns). Consequently, the reduction in the repetition frequency results in a reduction in the rate that optical pulses accumulate in the neuron. This, in turn, leads to fewer neural activations per time unit and directly links the incoming mean power with the number of generated excitations. This behavior enables a form of rate encoding scheme, in which inter-spike period encodes information related to the neuron’s input power^24^. The stability of the inter-spike period for a given stimulus, alongside the frequency variation of neural excitation for different stimuli, resembles the behavior of biological regular spiking neurons^46^,^59^.

An interesting implication that arises through the simultaneous existence of passive mode-locking (picosecond pulses) and neural excitation (nanosecond spikes) is that during each spike (Fig. 4d), the laser emits short optical pulses with picosecond duration (Fig. 3b,d). This emission pattern appears to be similar to bursting biological neurons^46^,^59^. The number of emitted pulses relates to the combination of the excitation duration (spike duration) and the repetition frequency of the laser. Therefore, the neuron can be designed to exhibit single or multi mode-locked pulse emission per excitation (spike).

**Figure 5:**
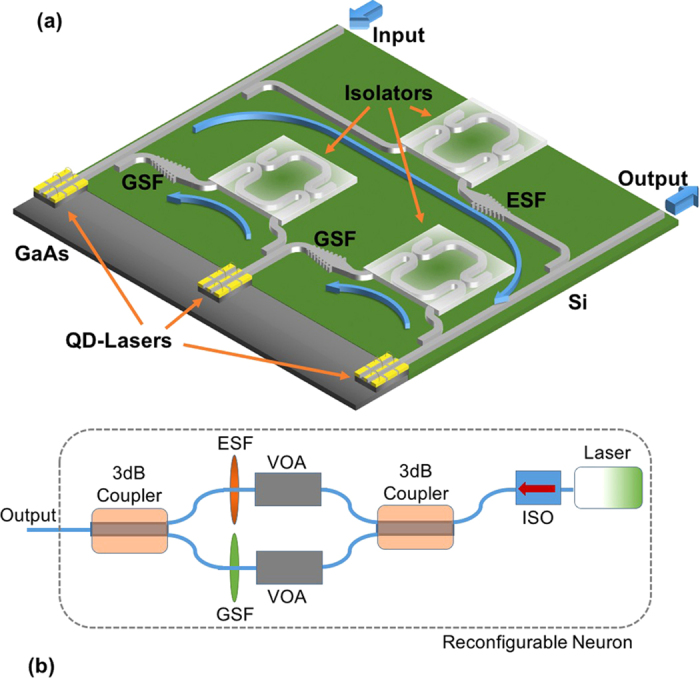
(**a**) Potential realization of a fully functional integrated artificial neural motif, similar to Fig. 1c. A passive/active integration technique can be used in order to allow the combination of III-V (quantum dot lasers) and IV materials (waveguides, filters, isolators). The terms GSF and ESF correspond to ground-state filter and excited-state filter respectively. (**b**) An alternative neuron design that enables neuron reconfiguration to achieve either excitatory or inhibitory operation with a simple adjustment of the bias at the saturable absorber and at the variable optical attenuator (VOA). ISO stands for integrated optical isolator.

The complementary operation of the two neurons can be combined to emulate a typical neural motif. As an example, in Fig. 5a, a potential integrated photonic circuit that enables similar operation to the neural motif present in Fig. 1c is demonstrated. It consists of three quantum-dot lasers in a hybrid integration scheme, in which wideband silicon-on-insulator chirped grating filters^60^ and integrated optical isolators based on first-order magneto-optical effects^61^ can be employed in order to allow full functionality. In detail, if a strong excitation signal is injected to the system’s input, then the first neuron is triggered to a firing activity (ground state pulses). That, in turn, can enable firing activity to the next in line node (ground state pulses). The output of this node is fed to the inhibition node, which is then also activated and starts emitting inhibitory signals (excited state pulses) to the first node, thus suppressing the initial activity. In addition, if a strong inhibition signal is injected to the input (excited state pulses) all the neurons are silenced^49^.

A desirable feature in neuro-mimetic systems is the ability to reconfigure the operational regime of each neuron and therefore to be able to realize different neural motifs with the same lasers. In our case, although the neuron core (laser) can be adjusted through applying a different voltage at the intracavity absorber, the existence of the waveband filters dictates an alternative setup to achieve re-configurability. In Fig. 5b such an approach is demonstrated. In addition to the optical isolator, a 3 dB coupler can be used at the laser’s output. At each coupler output a filter is placed (ground-excited state) accompanied by two voltage-driven variable optical attenuators. Through adjustment of the voltage of the laser and attenuator, the whole system can be tuned dynamically to inhibitory or excitatory operation.

Finally, yet importantly, it should be noted that the devices used in this work have a total cavity length of 2 mm, thus the total footprint is considerably large. However, mircodisk quantum-dot lasers with radii as short as 3 μm and low threshold currents are well within grasp^62^. Moreover, free carrier saturation and the associated waveband transitions, that are exploited in our case, are enhanced when the laser cavity is scaled down and consequently can be triggered with significantly lower injection current^63^, thus reducing the energy consumption of each neuron.

## Conclusion

The proposed scheme provides experimental evidence that an integrated quantum-dot passively mode locked laser can be used to realize both excitatory and inhibitory artificial neurons, through a simple adjustment of the built-in saturable-absorber’s reverse voltage, without the use of complex electro-optic schemes. The principle of operation in this scheme is based on the exploitation of the multi-band emission capabilities of quantum dot materials and the excitability effects of two section lasers. The emulation of both operational regimes and of frequency response effects offers a high degree of isomorphism to biological spiking neurons. Furthermore, the proposed scheme relies on widespread and mature fabrication technologies, whereas the all-optical realization of both regimes renders this scheme simpler to realize compared to electro-optic approaches. Consequently, this all-optical approach can pave the way for an integrated neuro-mimetic system that will be able to replicate complex neural functions and provide a novel computational paradigm for a variety of applications ranging from high speed sensory processing to adaptive control and robotics.

## Methods

### Quantum Dot Mode Locked Lasers

The devices used for the realization of the artificial neurons, were 2 mm long, consisting of two straight sections of 6 μm width. Each section had a physical isolation through a 1.4 μm deep etching. Electric characterization confirmed an electrical isolation of 1.5 KΩ. The laser structures were grown by molecular beam epitaxy on a GaAs <100> substrate and contain five self-assembled InAs/InGaAs quantum dot layers into 440 nm GaAs waveguide surrounded with Al_0.35_Ga_0.65_As claddings. The two facets of the devices are high reflection coated (95%)/anti-reflection coated (10%), respectively, for the operating wavelength (1275 nm). The devices under test were forced to a passive mode-locking operation by applying forward current in the gain section (85% per cent of total length), while the second section that was positioned near the high reflective facet was reverse biased, through a low noise voltage source. The repetition frequency of the devices was mainly governed by the total length of their cavities (20 GHz), while minor variations were recorded thanks to the different bias conditions employed. On the other hand, stronger variations (≈300 MHz) were measured when waveband switching occurred, due to the different refractive index of the material at each wavelength. A third device was used only as an input source for the dynamic response characterization of the neuron. The device consisted of 10 quantum dot layers and its length was 4 mm resulting in a repetition frequency of 10 GHz. The aforementioned fabrication procedure was followed in this case too.

### Injection Locking Experimental Setup

The experimental setup consists of a typical unidirectional injection-locking scheme. Optical isolators with a rejection ratio of 30 dB were placed after the master laser in order to prevent destabilization of the mode-locking process due to optical feedback effects and before the fiber interfaces of the optical instruments aiming to suppress the effect of optical feedback on the slave laser. Injection strength was adjusted through a polarization controller placed before the slave laser. Two waveband filters were used; the first was a long-pass filter with a cutoff wavelength at 1200 nm (20.5 dB rejection ratio), while the second was a short-pass wavelength filter with bandstop at 1200 nm (17.6 dB rejection ratio). The role of these filters was twofold; First to allow single waveband injection to the target neuron, and secondly to allow the monitoring of the power variation of each waveband separately. As mentioned above the gain section were biased through a low-noise current source, whereas the saturable absorber section was biased through a low-noise voltage source. The laser’s temperature was stabilized at 20 °C by means of a Peltier cooler in a closed control loop.

The measuring equipment consisted of a RF spectrum analyzer with optical bandwidth of 26.5 GHz that was used to monitor the electrical spectrum of the devices providing information for the repetition rate and the quality of the mode locking. An optical spectrum analyzer with a minimum resolution of 0.1 nm that was used to record the emitted optical spectrum, while a background free autocorrelator based on second harmonic generation with 10 fs resolution was used in order to acquire autocorrelation traces, assuming that the pulses had Gaussian shape.

## Additional Information

**How to cite this article**: Mesaritakis, C. *et al*. Artificial Neuron Based on Integrated Semiconductor Quantum Dot Mode-Locked Lasers. *Sci. Rep.* **6**, 39317; doi: 10.1038/srep39317 (2016).

**Publisher's note:** Springer Nature remains neutral with regard to jurisdictional claims in published maps and institutional affiliations.

## Supplementary Material

Supplementary Information

## References

[b1] Abu-Mostafa Y. S. & Psaltis D. Optical neural computers. Scientific American. 256(3), 88–95 (1987).

[b2] Modha D. S. . Cognitive computing. Commun. ACM. 54 **(8)**, 62–71 (2011).

[b3] Snider G. . From synapses to circuitry: Using memristive memory to explore the electronic brain. Computer. 44(2), 21–28 (2011).

[b4] Maass W. . Real-time computing without stable states: a new framework for neural computation based on perturbations. Neural Comput. 14(11), 2531–2560 (2002). 12433288 10.1162/089976602760407955

[b5] Tait A. N. . Photonic neuromorphic signal processing and computing. Nanophotonic Information Physics, Springer Berlin Heidelberg. 183–222 (2014).

[b6] Hasler J. & Marr B. Finding a roadmap to achieve large neuromorphic hardware systems. Front Neurosci. 10, 7–118 (2013). 10.3389/fnins.2013.00118 PMC3767911 24058330

[b7] Chu M. . Neuromorphic Hardware System for Visual Pattern Recognition With Memristor Array and CMOS Neuron. IEEE T. Ind. Electron. 62(4), 2410–2419 (2015).

[b8] Prodromakis T., Toumazou C. & Chua L. Two centuries of memristors. Nat. Material. 11, 478–481 (2012). 10.1038/nmat3338 22614504

[b9] Schemmel J., Fieres J. & Meier K. Wafer-scale integration of analog neural networks. IEEE Proc. Int. Jt. Conf. Neural Networks. 431–438, Hong Kong (2008).

[b10] Jin X. . Modeling spiking neural networks on SpiNNaker. Comput. Sci. Eng. 12(5), 91–97 (2010).

[b11] Arthur J. V. . Building block of a programmable neuromorphic substrate: A digital neurosynaptic core. IEEE Jt Conf. Neural Network. Brisbane. QLD 1–8 (2012).

[b12] Pruncal P. R. . Recent progress in semiconductor excitable lasers for photonic spike processing. Adv. Opt. Photonics. 8(2), 230–299 (2016).

[b13] Reed G. T., Mashanovich G., Gardes F. Y. & Thomson D. J. Silicon optical modulators. Nat. Phot. 4, 518–526 (2010).

[b14] Lee H., Chen T., Li J., Painter O. & Vahala K. J. Ultra-low-loss optical delay line on a silicon chip. Nat. Commun. 3, 867–873 (2012). 22643894 10.1038/ncomms1876

[b15] Brunner D. & Fischer I. Reconfigurable semiconductor laser networks based on diffractive coupling. Opt. lett. 40(16), 3854–3857 (2015). 26274677 10.1364/OL.40.003854

[b16] Nahmias M. A. . An integrated analog O/E/O link for multi-channel laser neurons. Appl. Phys. Lett. 108(15), 151106 (2016).

[b17] Mada H. Architecture for optical computing using holographic associative memories. Appl. Opt. 24, 2063–2066 (1985). 18223838 10.1364/ao.24.002063

[b18] Caulfield H. J. & Dolev S. Why future supercomputing requires optics. Nat Phot. 4, 261–263 (2010).

[b19] Nahmias M. A., Shastri B. J., Tait A. N. & Prucnal P. R. A Leaky Integrate-and-Fire Laser Neuron for Ultrafast Cognitive Computing. IEEE J. Sel. Top. Quantum Electron. 19(5), 1–12 (2013).

[b20] Kravtsov K., Fok M. P., Rosenbluth D. & Prucnal P. R. Ultrafast all-optical implementation of a leaky integrate-and-fire neuron. Opt. Express. 19, 2133–2147 (2011). 21369031 10.1364/OE.19.002133

[b21] Akiyama K. . A new Optical Neuron Device for all-Optical Neural Networks. Jpn. J. Appl. Phys. 30(12B), 3887–3892 (1991).

[b22] Poladian G. M. Reconfigurable optical neuron based on photoelectret materials. Appl. Optics. 39(5), 782–787 (2000). 10.1364/ao.39.000782 18337954

[b23] Izhikevich E. M. Neural excitability, spiking and bursting. Int. J. Bifurcation Chaos. 10, 1171–1266 (2000).

[b24] Shastri B. J. . Spike processing with a graphene excitable laser. Sci. Rep. 6, 19126–19138 (2016). 26753897 10.1038/srep19126 PMC4709573

[b25] Selmi F. . Relative refractory period in an excitable semiconductor laser. Phys. Rev. Lett. 112, 183902 (2014). 24856697 10.1103/PhysRevLett.112.183902

[b26] Larotonda M. A., Hnilo A., Mendez J. M. & Yacomotti A. M. Experimental investigation on excitability in a laser with a saturable absorber. Phys. Rev. A. 65(3), 033812 (2002).

[b27] Dubbeldam J. L. A., Krauskopf B. & Lenstra D. Excitability and coherence resonance in lasers with saturable absorber. Phys. Rev. E. 60, 6580–6588 (1999). 10.1103/physreve.60.6580 11970577

[b28] Plaza F. . Excitability following an avalanche-collapse process. Europhys. Lett. 38(2), 85–90 (1997).

[b29] Barbay S., Kuszelewicz R. & Yacomotti A. M. Excitability in a semiconductor laser with saturable absorber. Opt. Lett. 36, 4476–4478 (2011). 22139214 10.1364/OL.36.004476

[b30] Selmi F. ., Temporal summation in a neuromimetic micropillar laser. Opt. Lett. 40, 5690–5693 (2015). 26625083 10.1364/OL.40.005690

[b31] Coomans W. . Solitary and coupled semiconductor ring lasers as optical spiking neurons. Phys. Rev. E. 84, 036209 (2011). 10.1103/PhysRevE.84.036209 22060477

[b32] Van Vaerenbergh T. . Cascadable excitability in microrings. Opt. Express. 20, 20292–20308 (2012). 23037081 10.1364/OE.20.020292

[b33] Koen A. . Excitability in optically injected microdisk lasers with phase controlled excitatory and inhibitory response. Opt. Express. 21, 26182–26191 (2013). 24216842 10.1364/OE.21.026182

[b34] Goulding D. . Excitability in a quantum dot semiconductor laser with optical injection. Phys. Rev. Lett. 98, 4–7 (2007). 10.1103/PhysRevLett.98.153903 17501351

[b35] Yacomotti A. M. . Fast thermo-optical excitability in a two-dimensional photonic crystal. Phys. Rev. Lett. 97, 143904 (2006). 17155254 10.1103/PhysRevLett.97.143904

[b36] Brunstein M. . Excitability and self-pulsing in a photonic crystal nanocavity. Phys. Rev. A. 85, 031803 (2012).

[b37] Wieczorek S., Krauskopf B. & Lenstra D. Unifying view of bifurcations in a semiconductor laser subject to optical injection. Opt. Commun. 172, 279–295 (1999).

[b38] Garbin B. . Incoherent optical triggering of excitable pulses in an injection-locked semiconductor laser. Opt. Lett. 39, 1254–1257 (2014). 24690720 10.1364/OL.39.001254

[b39] Garbin B., Javaloyes J., Tissoni G. & Barland S. Topological solitons as addressable phase bits in a driven laser. Nat. Commun. 6, 5915 (2015). 25557181 10.1038/ncomms6915

[b40] Aragoneses . Unveiling the complex organization of recurrent patterns in spiking dynamical systems. Sci. Rep. 4, 4696–4712 (2014). 24732050 10.1038/srep04696 PMC3986700

[b41] Giudici M. . Andronov bifurcation and excitability in semiconductor lasers with optical feedback. Phys. Rev. E. 55, 6414–6418 (1997).

[b42] Wünsche H. J., Brox O., Radziunas M. & Henneberger F. Excitability of a semiconductor laser by a two-mode homoclinic bifurcation. Phys. Rev. Lett. 88, 023901 (2001). 11801013 10.1103/PhysRevLett.88.023901

[b43] Hurtado A. & Javaloyes J. Controllable spiking patterns in long-wavelength vertical cavity surface emitting lasers for neuromorphic photonics systems. Appl. Phys. Lett. 107, 241103 (2015).

[b44] Hurtado A., Henning I. D. & Adams M. J. Optical neuron using polarization switching in a 1550 nm-VCSEL. Opt. Express. 18, 25170– 25176 (2010). 21164863 10.1364/OE.18.025170

[b45] Hodgkin A. L. & Huxley A. F. A quantitative description of membrane current and its application to conduction and excitation in nerve. J. Physiol. 117(4), 500–544 (1952). 12991237 10.1113/jphysiol.1952.sp004764 PMC1392413

[b46] Markram H. . Interneurons of the neocortical inhibitory system. Nat. Rev. Neurosci. 5, 793–807 (2004). 15378039 10.1038/nrn1519

[b47] Paz J. T. & Huguenard J. R. Microcircuits and their interactions in epilepsy: is the focus out of focus? Nat. Neurosci. 18, 351–359 (2015). 25710837 10.1038/nn.3950 PMC4561622

[b48] Thivierge J. P. & Marcus G. F. The topographic brain: from neural connectivity to cognition, Trends Neurosci. 30(6), 251–259 (2007). 17462748 10.1016/j.tins.2007.04.004

[b49] Luo Liqun. Principles of Neurobiology. Garland Science (2015).

[b50] Shastri B. J. . SIMPEL: Circuit model for photonic spike processing laser neurons. Opt. Express. 23(6), 8029–8044 (2015). 25837141 10.1364/OE.23.008029

[b51] Rafailov E. U., Cataluna M. A. & Sibbett W. Mode-locked quantum-dot lasers. Nat. Phot. 1, 395–401 (2007).

[b52] Grillot F. . Optical feedback instabilities in a monolithic InAs/GaAs quantum dot passively mode-locked laser. Appl. Phys. Lett. 94(15), 153503 (2009).

[b53] Sugawara M. . Modeling room-temperature lasing spectra of 1.3-μm self-assembled InAs/GaAs quantum-dot lasers: Homogeneous broadening of optical gain under current injection. J. Appl. Phys. 97, 043523 (2005).

[b54] Mesaritakis C. . Effect of optical feedback to the ground and excited state emission of a passively mode locked quantum dot laser. Appl. Phys. Lett. 97, 061114 (2010).

[b55] Xu P. F. . Reduced linewidth enhancement factor due to excited state transition of quantum dot lasers. Opt. Lett. 37, 1298–1300 (2012). 22513665 10.1364/OL.37.001298

[b56] Kim J., Meuer C., Bimberg D. & Eisenstein G. Effect of Inhomogeneous Broadening on Gain and Phase Recovery of Quantum-Dot Semiconductor Optical Amplifiers. IEEE J. Quantum Electron. 46(11), 1670–1680 (2010).

[b57] Rohm A., Lingnau B. & Ludge K. Understanding Ground-State Quenching in Quantum-Dot Lasers. IEEE J. Quantum Electron. 51(1), 1–11 (2015).

[b58] Krauskopf B. . Excitability and self-pulsations near homoclinic bifurcations in semiconductor laser systems. Opt. Commun. 215(4), 367–379 (2003).

[b59] Izhikevich E. M. Simple Model of Spiking Neurons. IEEE T. Neural Networ. 14(6), 1569–1572 (2003). 10.1109/TNN.2003.820440 18244602

[b60] Simard Α. D. . Bandpass integrated Bragg gratings in silicon-on-insulator with well-controlled amplitude and phase responses. Opt. Lett. 40(5), 736–739 (2015). 25723420 10.1364/OL.40.000736

[b61] Shoji Y., Shirato Y. & Mizumoto T. Silicon Mach–Zehnder interferometer optical isolator having 8 nm bandwidth for over 20 dB isolation. JPN Appl. Phys. 53(2), 022202 (2014).

[b62] Mao M. H., Chien H. C., Hong J. Z. & Cheng C. Y. Room-temperature low-threshold current-injection InGaAs quantum-dot microdisk lasers with single-mode emission. Opt. Express. 19, 14145–14151 (2011). 21934777 10.1364/OE.19.014145

[b63] Markus A. . Simultaneous two-state lasing in quantum-dot lasers. Appl. Phys. Lett. 82, 1818–1820 (2003).

