# Supplementary material for: Artificial Neuron Based on Integrated Semiconductor Quantum Dot Mode-Locked Lasers
Source: Sci Rep. 2016 Dec 19;6:39317. doi: 10.1038/srep39317 (PMC5171909; doi:10.1038/srep39317)
Supplement: Supplementary Information [file srep39317-s1.doc]

# Artificial Neuron Based on Integrated Semiconductor Quantum Dot Mode-Locked Lasers

Charis Mesaritakis^a*^ , Alexandros Kapsalis^a^, Adonis Bogris^a,b^ and Dimitris Syvridis^a^

1. Department of Informatics & Telecommunications, National and Kapodistrian University of Athens, Panepistimiopolis Ilisia 15784, Athens, Greece
2. Department of Informatics, Technological Educational Institution of Athens, Aghiou Spiridonos, 12210 Egaleo, Athens, Greece

email*: cmesar@di.uoa.gr

Supplementary Information

Electro-Optic Characterization of the Devices

Prior to the optical neuron investigation all devices were electro-optically characterized utilizing part of the aforementioned setup. The 2mm long devices used for the realization of the optical neuron exhibited identical behaviour. The only difference between the devices, was the packaging. In the case of the slave laser (input source), light emitted was collected by means of a tapered fiber that resulted in a power coupling efficiency of only 10%. This value was evaluated by comparing the optical power measured through the taper and with a large area photodiode placed directly in front of the low reflective facet. On the other hand, the master laser (neuron) was in a fiber-pigtailed butterfly configuration and coupling efficiency was optimized. The 10 quantum dot layer device used during only the dynamic response characterization of the neuron, exhibited significantly different performance due to the variation in the cavity length (4mm) and in the number of active layers.

As shown in fig.1a-b the increase of the reverse voltage enhances the unsaturated losses and substantially reduces the emitted average power for both devices (2mm and 4mm). In the case of the 5 quantum dot layer device (fig. 1a) a significant decrease in the total emitted optical power is observed due to the onset of excited lasing (I_gain_=400mA). The same effect can be observed for the case of 4mm device (fig.1b) but it is less profound due to the increased current injection needed so as to trigger excited state lasing. This power reduction hinders the recording of excited state pulses in the autocorrelator, and in this case the evaluation of the mode locking quality is performed only through the RF spectrum. In fig. 1 c-d the pulse width for the ground state band is recorded for both types of devices. The narrowest pulse was in the order of 3ps, whereas in terms of peak power was 190mW. The evolution of pulse width for both cases versus the bias conditions is typical for such devices; in particular, the shortest pulses are recorded for high reverse voltage at the saturable absorber due to the increase of carrier recovery time, while limited injection current supresses self-saturation effects and enhances the pulse quality.


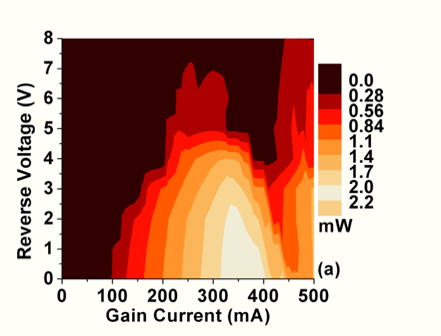

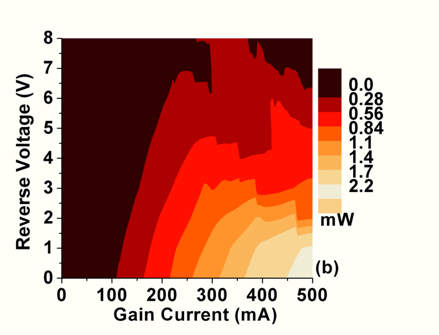


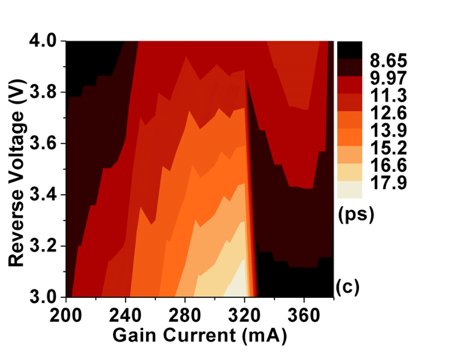

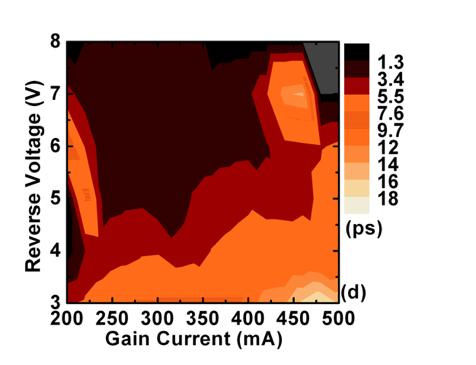


Figure 1. Average Power for the two laser structures (a): 5 QD layers 2mm long (b) 10 QD layers 4mm long, and the corresponding pulse width assuming Gaussian shape pulses.


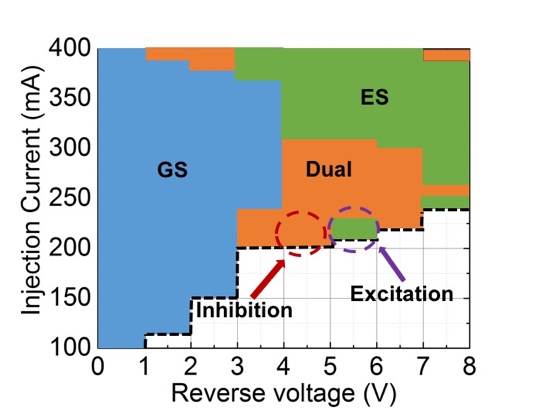


Fig. 2 Regimes of ground state (blue), excited state (green) and dual waveband emission (orange), versus the bias (voltage/gain current) for the 2mm device. The red circle corresponds to the Inhibition regime of operation, whereas the purple corresponds to the excitation regime.

The electro-optic characterization of the devices provided the best candidates for the realization of the optical neuron. In detail, five quantum dot layer devices exhibit increased gain saturation in the ground state and thus enable excited state mode locking through lower pumping current, while in the case of 10 or 15 quantum dot layer devices, excited emission is achieved only for pumping currents exceeding 400mA. In Fig. 2 a map depicting the different operational regimes for the 2mm long devices is presented where the two regimes (excitation/inhibition) are highlighted.
